# Indexing of grazing-incidence X-ray diffraction patterns: the case of fibre-textured thin films

**DOI:** 10.1107/S2053273318006629

**Published:** 2018-07-05

**Authors:** Josef Simbrunner, Clemens Simbrunner, Benedikt Schrode, Christian Röthel, Natalia Bedoya-Martinez, Ingo Salzmann, Roland Resel

**Affiliations:** a Department of Neuroradiology, Vascular and Interventional Radiology, Medical University Graz, Auenbruggerplatz 9, Graz, 8036, Austria; b E + E Elektronik Ges.m.b.H., Langwiesen 7, Engerwitzdorf, 4209, Austria; c Institute of Solid State Physics, Technical University Graz, Petersgasse 16, Graz, 8010, Austria; d Department of Physics, Department of Chemistry and Biochemistry, Concordia University, 7141 Sherbrooke Street W., SP 265-20, Montreal, Quebec H4B 1R6, Canada

**Keywords:** grazing-incidence X-ray diffraction, thin films, indexing, specular scan, mathematical crystallography

## Abstract

Crystal structure solutions from thin films are often performed by grazing-incidence X-ray diffraction (GIXD) experiments. In particular, on isotropic substrates the thin film crystallites grow in a fibre texture showing a well defined crystallographic plane oriented parallel to the substrate surface with random in-plane order of the microcrystallites forming the film. In the present work, analytical mathematical expressions are derived for indexing experimental diffraction patterns, a highly challenging task which hitherto mainly relied on trial-and-error approaches. The six lattice constants *a*, *b*, *c*, α, β and γ of the crystallographic unit cell are thereby determined, as well as the rotation parameters due to the unknown preferred orientation of the crystals with respect to the substrate surface. The mathematical analysis exploits a combination of GIXD data and information acquired by the specular X-ray diffraction. The presence of a sole specular diffraction peak series reveals fibre-textured growth with a crystallographic plane parallel to the substrate, which allows establishment of the Miller indices *u*, *v* and *w* as the rotation parameters. Mathematical expressions are derived which reduce the system of unknown parameters from the three- to the two-dimensional space. Thus, in the first part of the indexing routine, the integers *u* and *v* as well as the Laue indices *h* and *k* of the experimentally observed diffraction peaks are assigned by systematically varying the integer variables, and by calculating the three lattice parameters *a*, *b* and γ. Because of the symmetry of the derived equations, determining the missing parameters then becomes feasible: (i) *w* of the surface parallel plane, (ii) the Laue indices *l* of the diffraction peak and (iii) analogously the lattice constants *c*, α and ß. In a subsequent step, the reduced unit-cell geometry can be identified. Finally, the methodology is demonstrated by application to an example, indexing the diffraction pattern of a thin film of the organic semiconductor pentacene­quinone grown on the (0001) surface of highly oriented pyrolytic graphite. The preferred orientation of the crystallites, the lattice constants of the triclinic unit cell and finally, by molecular modelling, the full crystal structure solution of the as-yet-unknown polymorph of pentacene­quinone are determined.

## Abstract 2

Crystal structure solutions from fibre-textured crystals within thin films are frequently achieved by grazing-incidence X-ray diffraction experiments. In the present work, analytical mathematical expressions are derived for the indexing of experimental diffraction patterns.

## 1. Introduction   

The appearance of unknown polymorphs within organic thin films is a well known phenomenon which attracts considerable interest in organic electronics and pharmaceutical science (Jones *et al.*, 2016 ▸). Frequently used terms for this type of polymorph include substrate-induced phases, substrate-mediated phases or thin film phases (Bouchoms *et al.*, 1999 ▸; Schiefer *et al.*, 2007 ▸; Ehmann & Werzer, 2014 ▸). The presence of an isotropic substrate surface during the crystallization process can induce new types of molecular packing, because the substrate acts as a template for the crystallization process. Substrates on which such new polymorphs tend to grow typically exhibit a highly flat surface like oxidized silicon wafers, glass plates or polymer surfaces. There, the deposited organic material crystallizes with a strong preferred orientation showing a well defined crystallographic plane (the so-called contact, or texture plane) parallel to the substrate surface. However, no azimuthal (*i.e.* in-plane) order between the microcrystallites forming such films is observed due to the isotropic nature of the substrate surfaces. This type of crystalline orientation is called uniplanar texture (Heffelfinger & Burton, 1960 ▸) or fibre texture (Roe & Krigbaum, 1964 ▸).

**Figure 1:**
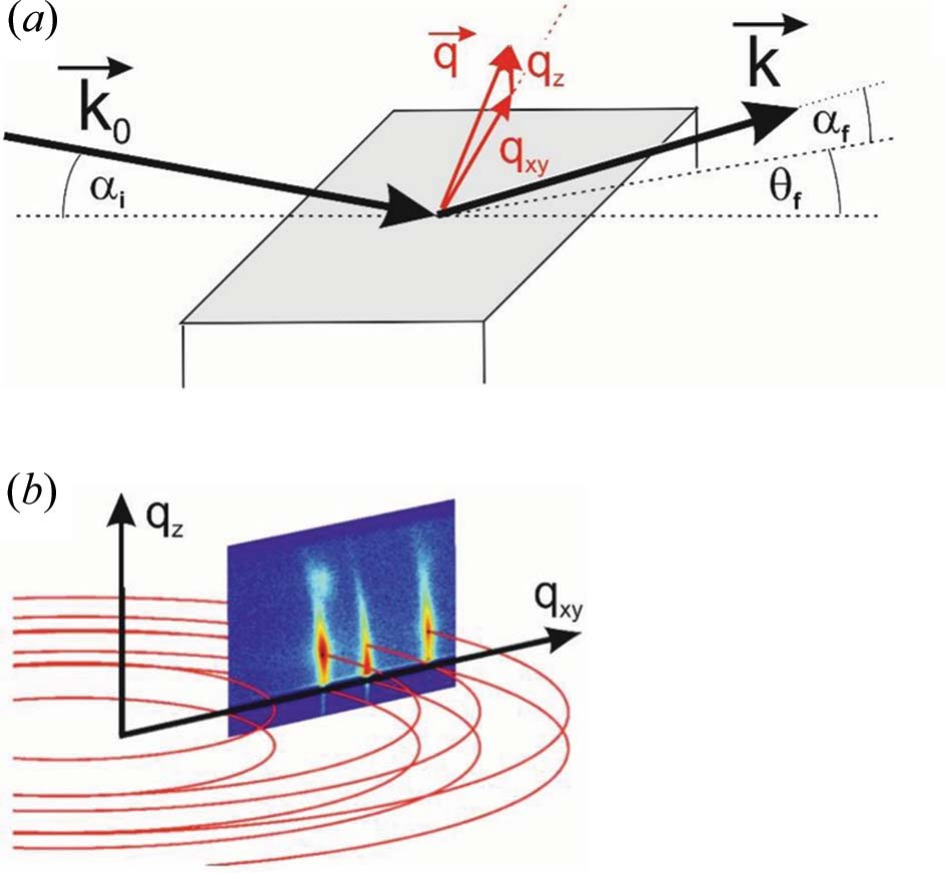
(*a*) Geometry of a grazing-incidence X-ray diffraction (GIXD) experiment with **k**_0_ and **k** representing the wavevectors of the primary and of the scattered X-ray beam, respectively, together with the corresponding angle of incidence α_i_, the in-plane scattering angle θ_f_ and the out-of-plane scattering angle α_f_. The corresponding scattering vector **q** is split into an in-plane part *q_xy_* and an out-of-plane part *q_z_*. (*b*) A reciprocal-space map measured in GIXD geometry plotted as a function of *q_xy_* and *q_z_* using a colour code for the measured intensity. The reciprocal-lattice points of the thin film crystallites grown in a fibre texture with a fibre axis oriented in the *z* direction degenerate to concentric rings around that axis.

Crystal structure solutions for such thin films are typically performed by grazing-incidence X-ray diffraction (GIXD); the experimental geometry is schematically shown in Fig. 1 ▸(*a*). The primary X-ray beam with the wavevector **k**_0_ and the scattered X-ray beam with the wavevector **k** determine the scattering vector **q** by **q** = **k** − **k**_0_. According to the Laue equation, diffraction occurs if the scattering vector **q** is equal to a reciprocal-lattice vector **g**. For organic crystallites in fibre-textured films, the reciprocal-lattice points lie on concentric circles, as illustrated by red circles in Fig. 1 ▸(*b*). Keeping the sample fixed in space, a GIXD experiment then equals a cut through the three-dimensional reciprocal space, roughly perpendicular to the rings of reciprocal-lattice points, and a corresponding two-dimensional reciprocal-space map is obtained [compare Fig. 1 ▸(*b*)]. Note that for thin films with defined in-plane alignment of the crystallites [*e.g.* if grown on anisotropic substrates like graphene (Salzmann *et al.*, 2012 ▸)] or for samples with weak statistics, the system can be artificially reduced to a fibre texture simply by a 360° rotation around the substrate normal (Röthel, 2017 ▸).

Crystal structure solutions from GIXD require the indexing of the diffraction pattern, that is, the assignment of Laue indices to the observed Bragg peaks. In our specific case of GIXD on fibre-textured films, two components of the reciprocal-lattice vectors – namely *q_z_* and *q_xy_* – are available for the indexing process (Smilgies & Blasini, 2007 ▸; Hailey *et al.*, 2014 ▸). This is considerably different to the indexing procedure employed for single-crystal diffraction patterns, where all three components of reciprocal-lattice vectors are recorded, as well as for powder diffraction of polycrystalline materials, where only the lengths of the scattering vectors are detected. In the case of single-crystal diffraction, three linearly independent reciprocal-lattice vectors are required to span the reciprocal lattice. Any other experimentally determined reciprocal-lattice vector has then to fit into this specific reciprocal lattice. Since complete three-dimensional vectors are used, even indexing of configurations with multiple lattices can be successfully achieved (Jacobson, 1976 ▸; Powell, 1999 ▸; Breiby *et al.*, 2008 ▸; Gildea *et al.*, 2014 ▸; Dejoie *et al.*, 2015 ▸; Morawiec, 2017 ▸). In the case of powder diffraction, only the lengths of the reciprocal-space vectors are used and the unknown variables are then up to six unit-cell parameters (in the case of a triclinic system) and a set of Laue indices with a triple of three integer values each. This problem cannot be solved algebraically. One possibility, however, is the dichotomy method where the cell constants are varied in increasingly smaller intervals and the *hkl* indices are subsequently refined using the least-square method (Boultif & Louër, 1991 ▸, 2004 ▸). For simplification of the indexing process, boundary conditions can be imposed.

Few programs have yet been developed for the indexing of two-dimensional reciprocal-space maps (Smilgies & Blasini, 2007 ▸; Breiby *et al.*, 2008 ▸; Hailey *et al.*, 2014 ▸; Jiang, 2015 ▸). Certainly, the situation is relatively trivial if all lattice parameters are known. However, for a successful indexing it is still necessary to determine the contact plane of the investigated crystals in fibre-textured films. For this reason, the rotation matrix of the thin film crystallites relative to the substrate surface has to be considered (Shmueli, 2006 ▸). If the lattice parameters are, however, unknown, *both* the lattice constants and the rotation matrix need to be determined, which represents a significantly more challenging task. Present approaches for the indexing of such systems are mainly based on trial and error, which is clearly unsatisfactory for obvious reasons.

Here, we demonstrate the analytical derivation of mathematical expressions to be employed in the indexing of two-dimensional reciprocal-space maps. To this end, we use two components of the reciprocal-space vectors, the in-plane part *q_xy_* and the out-of-plane part *q_z_*. A further input parameter for the indexing arises from specular X-ray diffraction experiments, as in essentially all cases of crystalline organic thin films grown in a fibre texture one defined Bragg peak (or one Bragg peak series) is observed at *q*_spec_, originating from the plane normal to the fibre axis of the film. This peak (series) is due to diffraction from the contact plane of the fibre-textured film, which is assigned to a crystallographic plane of Miller indices *u*, *v* and *w* (Salzmann & Resel, 2004 ▸; Smilgies & Blasini, 2007 ▸; Hailey *et al.*, 2014 ▸; Jiang, 2015 ▸). By combining the peak positions in the GIXD pattern (*q_xy_*, *q_z_*) with the specular peak (*q*_spec_), the required number of unknown parameters for indexing significantly reduces.

If all three components of the scattering vectors are measured, the orientation of the crystal has to be considered by including the rotation parameters. Though the number of equations is smaller than the number of unknowns, the analytical treatment is much more straightforward since it is purely based on linear equations (see Appendix *E* [App appe]).

## 2. Methods   

For the following mathematical treatise a laboratory coordinate system with the *xy* plane being parallel to the substrate surface is assumed.

### 2.1. Non-rotated case – contact plane (001)   

In the following analysis, *a*, *b*, *c*, α, β and γ are the parameters of the (direct) unit cell, and a*, b*, c*, α*, β* and γ* are the reciprocal cell parameters (Giacovazzo, 2011 ▸), which are summarized in Table 1 ▸.

If the (001) lattice plane is parallel to the substrate surface in a GIXD experiment, the reciprocal-lattice vector **g** with its Laue indices *h*, *k* and *l* can be represented by the equation
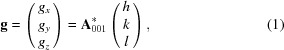
where the matrix 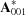 is given as
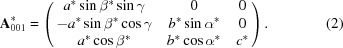
When the Laue condition **q** = **g** is fulfilled, diffraction can be observed.

In the real space, **A**_001_ characterizes the matrix of the lattice vectors **a**_0_, **b**_0_ and **c**_0_, which is in the non-rotated system given by 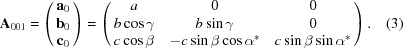
Equations (2) and (3) are connected *via*

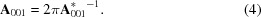
The volume *V* of the unit cell can be calculated by 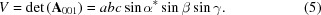
Using equation (1) and the relations given in Table 1 ▸, the in- and out-of-plane components of the reciprocal vector **g** can be explicitly written as 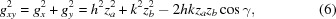


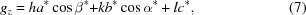
with 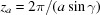 and 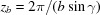. From equation (6) the unit-cell parameters which are oriented in-plane, namely *a*, *b* and γ, can be determined; in further consequence equation (7) leads to parameters *c*, α and β. The integer variables of the Laue indices have to be varied and the values of *q_xy_* and *q_z_* from three independent Bragg peak series are required to obtain a solution for the corresponding unit-cell parameters, which have to be checked if proper Laue indices can be obtained for all measured diffraction peaks (Truger *et al.*, 2016 ▸).

### 2.2. Rotated case – contact plane (*uvw*)   

**Figure 2:**
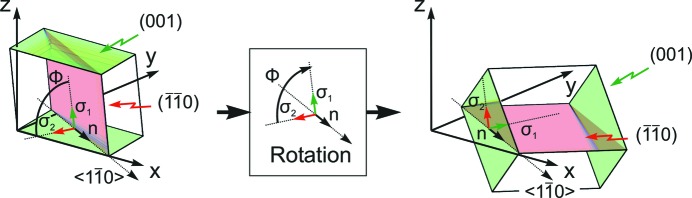
Sketch of a triclinic crystal cell oriented with its (001) net plane parallel to the *xy* plane. For studying more general orientations with, *e.g.*, a (−1−10) contact plane, all planes and vectors have to be rotated around the zone axis [1−10] by the angle Φ. The zone axis is defined by the vector **n**, being orthogonal to **σ**_1_ and **σ**_2_. As visualized in the right part of the figure, lattice planes as well as crystallographic directions follow this transformation.

Obviously, the situation becomes more complex if the (001) lattice plane is *not* parallel to the substrate surface as the matrix 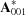 has now to be transformed. In particular, it has to be rotated around the zone axis which is defined by the (001) plane (characterized by its normal vector **σ**_1_) and the new contact plane (*uvw*), as characterized by its normal vector **σ**_2_. A graphical sketch of the discussed geometry is presented in Fig. 2 ▸.

It can easily be proven that an arbitrary rotation of the lattice vectors in the real space corresponds to an identical rotation of the reciprocal-space vectors. If **R** is an arbitrarily chosen rotation matrix acting on the lattice vectors and **R** ^−1^ = **R** ^T^ is its inverse, the following relation can be deduced from equation (4): 
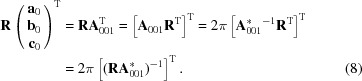
Therefore, equation (4) can be generalized and written in the form 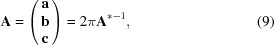
where **a** = **Ra**_0_, **b** = **Rb**_0_ and **c** = **Rc**_0_ are the rotated lattice vectors and 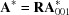.

Based on the graphical representation it can be shown that the unit vector **n** of the zone axis is calculated by the vector product of **σ**_1_ and **σ**_2_: 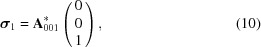


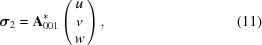


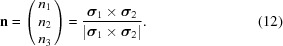



The angle of rotation 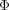 is obtained by the scalar product 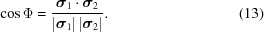



The matrix **R**, which describes a rotation by 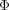 around the axis **n**, is given by (Shmueli, 2006 ▸) 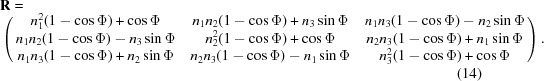
Combining equations (10) to (12) yields the components of the zone axis unit vector **n**: 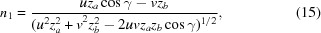


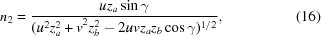


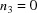
, which results in the condition 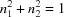. In a next step the angle of rotation Φ can be obtained by combining equations (10), (11) and (13) as 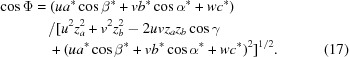



Finally, the reciprocal-lattice vector **g** can be written as 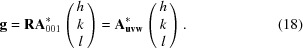
From equation (18), the following expressions for the radius *g_xyz_* 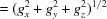 and the out-of-plane part *g_z_* of the reciprocal-lattice vector can be derived: 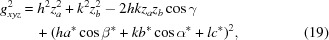


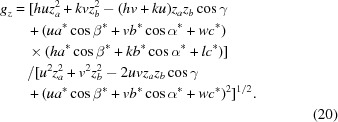
If the condition 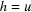, 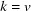 and 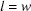 is fulfilled, equations (19) and (20) are identical, which means that there is only a contribution from the out-of-plane part *g_z_*, whereas the in-plane part *g_xy_* is zero. This is valid for the specular scan *g*_spec_, which is exactly sensitive to the lattice plane parallel to the surface, and therefore can be explicitly written as 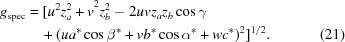



From equations (19) to (21) and by including equation (5), the following expression for the in-plane part *g_xy_* can be derived: 
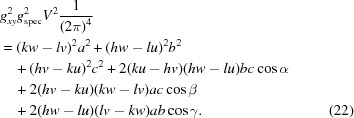
Furthermore, using equations (20) and (21), equation (19) can be rewritten as 
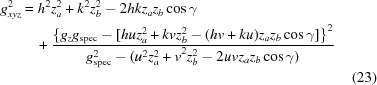
and by algebraic transformations the following expression can be derived: 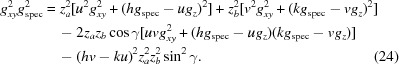
Equation (24) can be regarded as a generalization of equation (6), additionally including the two rotational integer parameters *u* and *v*, the specular scan *g*_spec_ and the out-of-plane part *g_z_*. For *u* = *v* = 0 it reduces to equation (6) in the non-rotated case.

Equation (24) comprises – in addition to the rotation parameters *u* and *v* – only the lattice parameters *a*, *b*, γ and the Laue indices *h* and *k*. This facilitates the mathematical analysis, where the integer variables can be varied and only three real unknowns have to be calculated. Therefore, we note that when indexing GIXD patterns, the acquisition of a specular scan is of considerable help.

In rare cases, net planes oriented parallel to the substrate surface are characterized by a weak structure factor which inhibits the acquisition of a specular scan (Djuric *et al.*, 2012 ▸). In such cases *u* and *v* must be assumed to be real (instead of integer) numbers which makes the mathematical analysis more exhaustive and an alternative notation of the rotation matrix may be chosen (see Appendix *A* [App appa]).

In Table 2 ▸ we provide a summary of the derived equations and further analogous expressions due to symmetry relationships.

As the Laue indices are integers, they can be systematically varied, whereas the real unknown parameters *a*, *b* and γ can be calculated from the *q_xy_* and *q*_*z*_ values of three independent Bragg peak series. This can be achieved analytically by employing proper mathematical substitutions to obtain linear equations (see Appendix *B* [App appb]).

For calculating the remaining cell parameters from equations (20) and (21) the following expression can be derived: 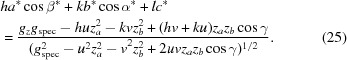
Equation (25) can be regarded as a generalization of equation (7) to which it reduces for *u* = *v* = 0 in the non-rotated case.

Alternatively, by using the symmetry expressions in Table 2 ▸ the parameter sets {*a*, *c*, β, *u*, *w*} and {*b*, *c*, α, *v*, *w*} can be determined in an analogous manner as {*a*, *b*, γ, *u*, *v*}.

If one component of the zone axis, which is the intersection of the planes (*uvw*) and (*hkl*), is zero, compact expressions for the reciprocal cell parameters a*, b*and c* can be derived (see Appendix *C* [App appc]).

## 3. Discussion – determining the reduced cell   

As discussed by Niggli, the reduced cell is defined by the cell that satisfies the conditions derived from the reduction theory of quadratic forms (Niggli, 1928 ▸). Such a cell provides a unique description of the lattice and is characterized independently of lattice symmetry. The main conditions for reduction require that the unit cell is based on the three shortest vectors of the lattice; such a unit cell is then called a Buerger cell (Buerger, 1957 ▸). However, this cell may not be unique. An unambiguous unit cell is the so-called reduced cell defined by Niggli (Niggli, 1928 ▸; Santoro & Mighell, 1970 ▸). The general criteria for the reduced cells are summarized in Table 3 ▸; the complete criteria, which include special conditions, are listed in the *International Tables of Crystallography* (De Wolff, 2016 ▸).

If **a**, **b** and **c** are the lattice vectors of the reduced cell, then every linear combination **a**′, **b**′ and **c**′ of these vectors
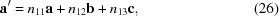


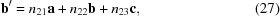


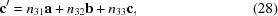
where 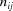 are integers and components of the transformation matrix 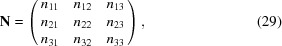
can be regarded as a superlattice, which obeys the Laue condition (Santoro *et al.*, 1980 ▸). Therefore, in general, any solution that is found when indexing a diffraction pattern must be analysed if it satisfies the conditions of the reduced cell.

In the matrix approach to symmetry (Himes & Mighell, 1987 ▸) **N** is represented by one of the 64 symmetry matrices to check if the transformation leads to identity (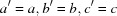, α′ = α, β′ = β, γ′ = γ).

Equations (26) to (28) can be equivalently written as **A**′ = (**a**′, **b**′, **c**′)^T^ = **NA**. Considering equation (9) the following relations are valid:
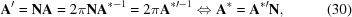


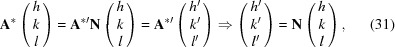
where *h*′, *k*′ and *l*′ are the Laue indices in the transformed system. Thus the transformation **N** which converts the lattice vectors is the same as that which converts the Laue indices in the reciprocal space. This is summarized in Table 4 ▸.

Therefore, reduction of the cell parameters to the reduced cell (Santoro & Mighell, 1970 ▸; Mighell, 1976 ▸; Křivý & Gruber, 1976 ▸) is equivalent to converting the Laue indices as in the common reciprocal metric tensor approach (Kroll *et al.*, 2011 ▸). If there are two solutions to a diffraction pattern with the Laue indices 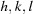 of the unitary cell and 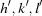 of a superlattice, the transformation matrix **N** can be easily obtained by linearly independent Laue indices of three reflections:
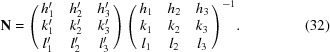
The Miller indices *u*, *v* and *w* can equally be used. If the determinant of the transformation matrix equals ±1, the cell volume does not change. Thus, systematically combining three linearly independent triples of Laue indices, respectively, and calculating their determinants can give an estimate of whether a found solution may match the Buerger cell. Furthermore, in GIXD, after finding a set of cell parameters, by calculating three linearly independent reciprocal vectors and evaluating their inverse matrix the three shortest lattice vectors can be determined (see Appendix *E* [App appe]).

The criteria for reduced cells demand that 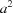 ≤ 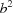 ≤ 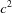 and that the angles are either acute (type I) or obtuse (type II). For this, the expressions in Table 5 ▸, which directly result from the symmetric properties of the equations in Table 2 ▸, are helpful.

## 4. Example: pentacene­quinone on highly oriented pyrolytic graphite   

We now employ our novel formalism in the indexing of a thin film of 6,13-pentacene­quinone (PQ, C_22_H_12_O_2_), which was grown on a freshly cleaved, highly oriented pyrolytic graphite (HOPG) substrate by physical vapour deposition under high vacuum conditions (base pressure <5 × 10^−6^ Pa; deposition rate 0.5 nm min^−1^; final nominal film thickness 30 nm, as determined by a quartz crystal microbalance). The film was then investigated at the beamline W1 at the synchrotron radiation source DORIS (DESY, HASYLAB, Germany). GIXD experiments together with specular X-ray diffraction were performed using a goniometer in pseudo 2+2 geometry by a one-dimensional detector (MYTHEN, Dectris) and a wavelength of 1.1796 Å for the primary X-ray beam. The specular scan was performed in the 2θ range of 2° (*q_z_* = 0.185 Å^−1^) to 26° (2.395 Å^−1^). For the GIXD experiments, the incident angle of the primary beam was set to α_i_ = 0.13°. The in-plane scattering angle θ_f_ was varied between 3° and 40° in steps of 0.05° where for every step an out-of-plane scattering range of Δα_f_ = 3.5° was recorded. In total, seven scans along θ_f_ were performed so that the complete covered angular range of α_f_ was 0° to 24.5°. The diffraction pattern was transformed from real to reciprocal space using the custom-made software *PyGID* (Moser, 2012 ▸). The resulting reciprocal-space map illustrates measured intensities on a logarithmic scale by a colour code. The exact positions of the Bragg peaks in terms of *q_xy_* and *q_z_* were determined by integration of the intensities along *q_xy_* and *q_z_*, respectively, and fitted by Gaussian curves. The *q_z_* values of the peak positions were corrected in terms of refraction effects; a maximum variation of 0.011 Å^−1^ was obtained (Resel *et al.*, 2016 ▸).

**Figure 3:**
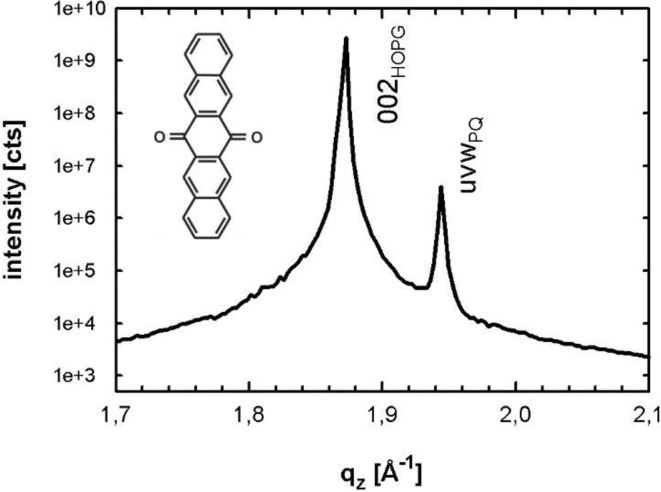
Specular X-ray diffraction of a crystalline thin film of PQ grown on HOPG. The inset gives the chemical structure of the molecule.

**Figure 4:**
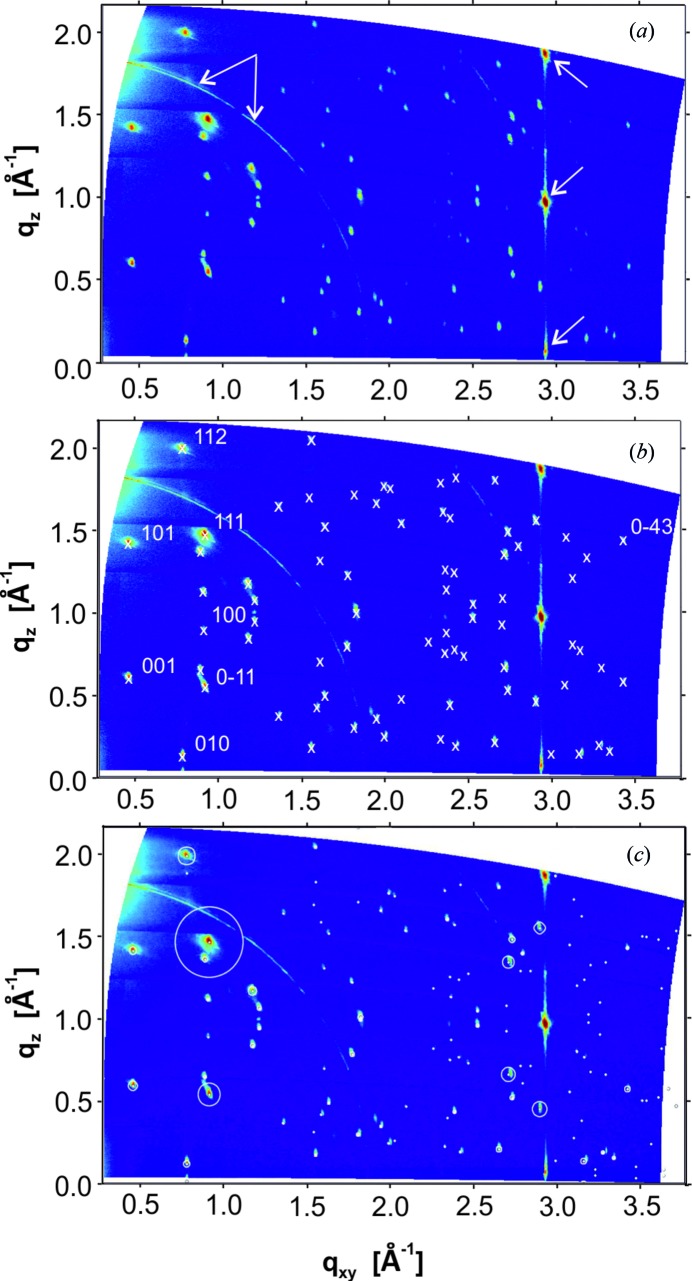
(*a*) Reciprocal-space map (RSM) of the PQ thin film grown on HOPG (*cf*. Fig. 3 ▸); arrows indicate diffraction features of the substrate. (*b*) Indexing of the RSM. Crosses denote calculated peak positions assigned to experimentally observed peaks; for clarity, Laue indices are given only for selected Bragg peaks. (*c*) RSM with calculated peak intensities obtained from the theoretically determined molecular packing; the area of the circles corresponds to the square of the structure factors.

Fig. 3 ▸ shows the specular diffraction pattern where only the region around the two dominant diffraction peaks is depicted. The peak at *q_z_* = 1.873 Å^−1^ (*d* = 3.355 Å) agrees well with the expected peak position of the 002 reflection of graphite (*d* = 3.354 Å) based on the lattice constants of *a* = 2.459 and *c* = 6.708 Å (Baskin & Meyer, 1955 ▸). The second peak located at *q*_spec_ = 1.946 Å^−1^ (*d* = 3.229 Å) is assigned to the PQ crystals. Fig. 4 ▸(*a*) shows the diffraction pattern of the GIXD experiment. Bragg peaks at *q_xy_* = 2.946 Å^−1^ and *q_z_* = 0.002 Å^−1^, *q_z_* = 0.941 Å^−1^ and *q_z_* = 1.880 Å^−1^ are identified as the 10−10, 10−11 and 10−12 reflections of the HOPG single-crystal substrate. Additionally, a Debye–Scherrer ring appears at *q* = 1.87 Å^−1^ which is assigned to disordered 0002 planes of graphite. The diffraction features of the HOPG substrate are marked by arrows in Fig. 4 ▸(*a*).

The remaining Bragg peaks are assigned to PQ crystals; they are distributed within the whole reciprocal-space map. The most intense peaks with their *q_xy_* and *q_z_* positions were used together with *q*_spec_ = 1.946 Å^−1^ for the indexing routine. A total of 74 reflections of the GIXD map were included in the analysis. In a first step of indexing the Miller indices *u* and *v* of the contact plane (the crystallographic plane which is parallel to the substrate surface) are varied by integer variables together with a systematic change of the Laue indices of three reflections so that a first set of lattice constants *a*, *b* and γ are obtained [see equation (43) in Appendix *B* [App appb]]. Note that with the restriction of linear independency due to linear transformation [see equation (32)] three pairs of Laue/Miller indices are, in principle, freely eligible to get a mathematically valid solution which may represent a superlattice. This first set of lattice constants is used to determine the Laue indices *h* and *k* of all other peak positions (*q_xy_*, *q_z_*) until a suitable assignment of the Laue indices *h* and *k* to all 74 reflections is obtained. For this procedure, mathematical expressions in Appendix *D* [App appd] are helpful. In total 150 integer variables and three real numbers have to be determined.

In a subsequent step, the assignment of the remaining Miller index *w* as well as the Laue indices *l* and the evaluation of the lattice constants *c*, α and β have to be accomplished. There are two possible ways. The first possibility relies on symmetry considerations of equation (24) (see Table 2 ▸). A systematic exchange of the two Miller indices, the pairs of Laue indices and the three lattice constants leads to a set of three equivalent equations where, finally, all parameters of the indexing are determined. The second possibility is simply using equations (21) and (25), where the remaining integer *w* of the contact plane, the lattice constants *c*, α and β as well as the Laue indices *l* of the 74 reflections are obtained. Expressions in Appendix *C* [App appc] can be helpful in determining the parameters c*, *w* and *l* of specific reflections. In a last step, when all integer variables have been assigned, the values of the real lattice parameters can be fitted. For this procedure expressions in Table 2 ▸ can be used.

As the underlying equations do not allow a unique mathematical solution, a manifold of possible results exist. But crystallographic restrictions constrain these mathematical solutions. The cell parameters must obey the scalar product (Niggli) criteria (see Table 3 ▸). Furthermore, one has to check if a solution has the shortest possible edges and thus is a Buerger cell.

For illustration, we depict the following two mathematical solutions, both of which obey the scalar product criteria for type-II cells:

Solution 1: *u*_1_ = 1, *v*_1_ = 0, *w*_1_ = 2; *a*_1_ = 5.067 Å, *b*_1_ = 8.064 Å, *c*_1_ = 8.882 Å, α_1_ = 91.64°, β_1_ = 93.34°, γ_1_ = 94.01°, *V*_1_ = 361.2 Å^3^.

Solution 2: *u*_2_ = 1, *v*_2_ = 2, *w*_2_ = −2; *a*_2_ = 5.067 Å, *b*_2_ = 11.824 Å, *c*_2_ = 12.166 Å, α_2_ = 95.53°, β_2_ = 90.22°, γ_2_ = 95.25°, *V*_2_ = 722.4 Å^3^.

In Table 6 ▸, corresponding Laue triples of some lower reflections are given. The determinants of three linearly independent triples of indices give mostly ±1 for solution 1 and ±2 for solution 2. The transformation matrix **N** which leads from solution 2 to solution 1 can be determined according to equation (32):
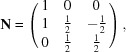
with det(**N**) = ½, establishing the relations between the lattice parameters, the Miller indices of the contact plane and the Laue indices of the 74 Bragg peaks (see Table 3 ▸).

By applying equation (18), three reciprocal-lattice vectors **g**, *e.g.* of the three Laue triples (1,0,1), (0,1,1) and (0,1,0) for solution 1, and (1,1,−1), (0,2,0) and (0,1,1) for solution 2, can be calculated. The determinants of the vector matrices should be as small as possible but not equal to zero. By determining their inverse matrices and by multiplying these with vectors **m** = 2π(*m*_1_, *m*_2_, *m*_3_)^T^, where *m_i_* are systematically varied integers between −2 and 2, lattice vectors can be obtained [see Appendix *E* [App appe] with emphasis on equation (71)]. In both cases, listing the lengths of these vectors in ascending order yields 5.067, 8.064, 8.882, 9.219, 9.819, 9.966, 10.134, 10.479, 11.824 and 12.166 for the ten shortest vectors. The *z* components of these vectors are all integer multiples of 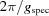 (in absolute values 1, 0, 2, 1, 1, 3, 2, 1, 2, 2, respectively, and thus representing the Miller indices – see the equations in Table 7 ▸). Therefore, solution 1 matches the reduced cell, whereas solution 2 represents a superlattice. The thus obtained vectors of both solutions, though they do not coincide, but are equally rotated, span identical parallelepipeds and result in the same cell parameters *a*, *b*, *c*, α, β and γ. Therefore, solution 2 can be reduced very effectively by the described method.

For evaluating the reliability of powder pattern indexing, a factor *F_N_* has been introduced (Smith & Snyder, 1979 ▸). For GIXD we suggest the following factors for assessing the accuracy of the obtained result:
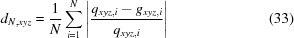


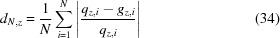
where *N* is the number of reflections, (*q_xyz,i_*, *q_z,i_*) are the measured and (*g_xyz,i_*, *g_z,i_*) are the calculated peak positions of the *i*th reflection. In our case *d*_74,*xyz*_ = 0.0022 and *d*_74,*z*_ = 0.0032. However, it should be emphasized that it is additionally necessary to prove that the obtained unit cell corresponds to the reduced cell.

Since the unit-cell dimensions are considerably different to the three reported phases of PQ (Dzyabchenko *et al.*, 1979 ▸; Nam *et al.*, 2010 ▸; Salzmann *et al.*, 2011 ▸), we can conclude that a new polymorph is found. Based on the reduced cell the peak positions are calculated and plotted in Fig. 4 ▸(*b*). A total of 80 positions of Bragg peaks could be assigned to PQ crystals by their Laue indices.

If the specular scan is not known, it is then an additional unknown parameter in equation (24), which has to be solved numerically by using four pairs of input parameters *q_xy_* and *q_z_*. An alternative way would be to exclude the specular diffraction peak from the indexing procedure: an alternative notation of the rotation matrix may then be used [see equation (35) in Appendix *A* [App appa]]. Even in that case a two-step separation of the indexing can be obtained. Input parameters are the total length of the scattering vectors *q_xyz_* and *q_z_* and the estimated parameters are the lattice constants *a*, *b*, γ and the two angles ψ and φ which express the orientation of the crystal at the substrate surface [equation (38)]. Note that *q_xyz_* can be easily determined by 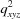 = 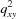 + 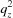 and due to the Laue condition *q_xyz_* = *g_xyz_* and *q_z_* = *g_z_*. In our case the rotation angles ψ = 94.01° and φ = 39.78° are obtained. The other lattice constants *c*, α and β can be obtained from equation (37).

There are different possibilities to determine the molecular packing based on the knowledge of the crystallographic unit cell (David *et al.*, 2006 ▸). In the case of organic thin films rigid-body refinement procedures based on experimental structure factors were used (Krauss *et al.*, 2008 ▸; Mannsfeld *et al.*, 2011 ▸) or theoretical modelling was applied (Schiefer *et al.*, 2007 ▸; Jones *et al.*, 2017 ▸). Here, the molecular packing relative to the experimentally determined unit cell has been determined by theoretical modelling, where a combination of molecular dynamics (MD) simulations and density functional theory (DFT) was used. MD simulations were carried out using the *LAMMPS* code in combination with the *CHARMM General Force Field* v.2b7 (Plimpton, 1995 ▸; Vanommeslaeghe *et al.*, 2010 ▸). In a first step, several hundred trial structures were created by placing one molecule randomly into a slightly expanded unit cell. During the subsequent MD run, the system was allowed to relax energetically while the unit cell was continuously shrinking to the experimental size. The most promising structures were further redefined using DFT geometry optimizations as implemented in the *VASP* package (version 5.4.1) (Kresse & Hafner, 1993 ▸, 1994 ▸; Kresse & Furthmüller, 1996*a* ▸,*b* ▸). The Perdew–Burke–Ernzerhof functional for the exchange and correlation (Perdew *et al.*, 1996 ▸) and projector-augmented wave potentials for all the elements (Blöchl, 1994 ▸; Kresse & Joubert, 1999 ▸) were used. Van der Waals corrections were included following the many-body dispersion approach of Tkatchenko *et al.* (2012 ▸). A plane-wave cut-off energy of 800 eV and a converged Monkhorst–Pack grid (Monkhorst & Pack, 1976 ▸) of 7 × 4 × 4 were used. The total energy during the self-consistency loop of each DFT step was converged to 10^−8^ eV. Calculations were performed using the experimental volume, relaxing the atomic positions down to a threshold of 10^−3^ eV Å^−1^ on forces. Based on the molecular packing a diffraction pattern was calculated. The result is depicted in Fig. 4 ▸(*c*), where the intensity as well as the position of the Bragg peaks are illustrated by circles. The centre of the circles gives the peak position, while the area of the circles gives the square of the corresponding structure factors. An excellent agreement between experimentally and calculated peak intensities is found; hence, the resulting molecular packing describes the surface-induced phase of PQ on HOPG. The CIF for the solved crystal structure can be found in the supporting information.

**Figure 5:**
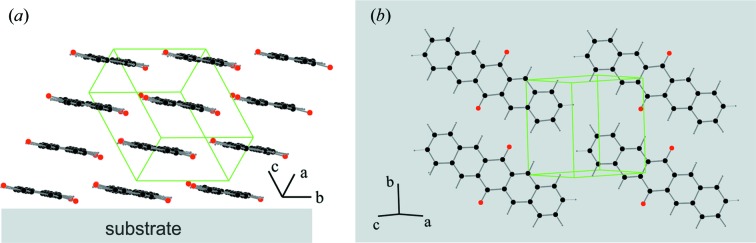
Molecular packing of PQ molecules as well as orientation of the molecules relative to the substrate surface determined from the crystal structure solution: in a side view along the long molecular axis (*a*) and in a top view of a single molecular layer across the crystallographic (102) plane (*b*). The crystallographic unit cell is depicted in green.

The packing of the PQ molecules within the crystal structure can be described by a parallel stacking of the planar molecules. The stacking distance between the planar molecules is about 3.45 Å (Fig. 5 ▸ *a*). Short contacts appear between O atoms and neighbouring H atoms and between terminal H atoms of neighbouring PQ molecules (Fig. 5 ▸ *b*). In a subsequent step the orientation of the molecules relative to the substrate surface can be determined. The described indexing routine reveals the assignment of the Laue indices 102 to the specular diffraction. Plotting the crystallographic plane with Miller indices 102 towards the molecular packing of our crystal structure solution directly reveals the orientation of the molecules relative to the substrate surface (Fig. 5 ▸ *a*). It is found that the long molecular axes are aligned parallel to the substrate surface. The molecular plane encloses an angle of 8° to a ‘flat-on’ orientation. It seems that the enhanced intermolecular interactions *via* the short oxygen–hydrogen bonds (discussed above) establish a stabilization of a layer formed by tilted molecules.

## 5. Conclusion   

In the present work, we provide a unifying framework for the indexing of reciprocal-space maps obtained by GIXD on fibre-textured thin films, which we successfully apply in deriving the full structure solution of an as-yet-unknown substrate-mediated polymorph of PQ.

Including the specular peak in the mathematical formalism of diffraction experiments can be of considerable help, especially in the case of GIXD where the spatial orientation of the unit cell has to be considered. For the rotation parameters the integer variables *u*, *v* and *w* can be employed, and mathematical expressions can be derived in which the unknown cell parameters are considerably reduced. This significantly reduces computational efforts, as the integer variables can be systematically varied and only three real unknown parameters remain, which can be analytically calculated using *q_xy_* and *q_z_* of three independent diffraction peaks. In subsequent steps the remaining parameters can then be conveniently determined.

As any linear combination of the unit-cell vectors satisfies the imposed mathematical conditions no unique solution exists. Based on the well known criteria originally imposed by Niggli, the reduced unit cell has therefore to be determined. The main conditions for reduction require that the cell is based on the three shortest vectors of the lattice. These can be obtained from any mathematical solution by the proper three-dimensional linear transformation. It may be helpful to use the obtained cell parameters and Laue indices to calculate three linearly independent reciprocal vectors and evaluate their inverse matrix to determine the lattice vectors of the reduced unit cell.

Though our analysis primarily considers the general case of a triclinic system, it also applies to crystal systems of higher symmetries which then imply a higher impact of symmetry considerations such as that of reflection conditions.

## Supplementary Material

Crystal structure: contains datablock(s) Pentacenequinone. DOI: 10.1107/S2053273318006629/wo5026sup1.cif

## Figures and Tables

**Table 1:** Relations between the parameters of the direct lattice (*a*, *b*, *c*, α, β, γ) and of the reciprocal lattice (a*, b*, c*, α*, β*, γ*) and the volume of the crystallographic unit cell *V*

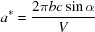	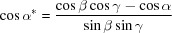	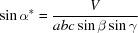
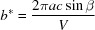	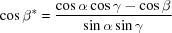	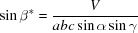
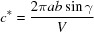	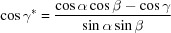	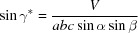
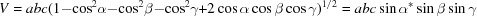		
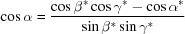	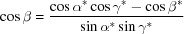	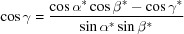

**Table 2:** Relations for the total length *g_xyz_*, the out-of-plane part *g_z_* and the in-plane part *g_xy_* of the reciprocal-space vectors with indices *hkl* and of the vector *uvw* (*g*_spec_) by using direct- and reciprocal-lattice parameters and the volume *V*

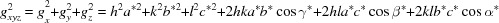	
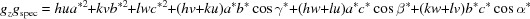	
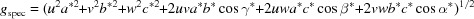	
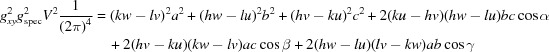	
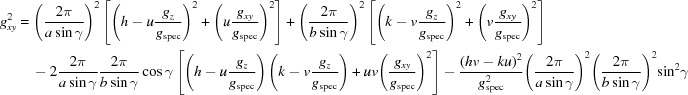	
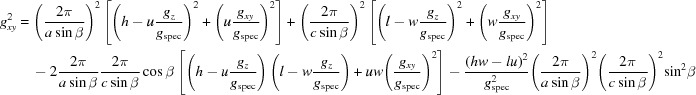	
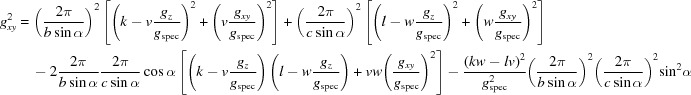	

**Table 3:** General criteria for reduced cells

General criteria for reduced cell type I (positive reduced cell; all of the angles are <90°)†	
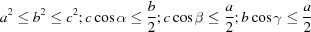	
General criteria for reduced cell type II (negative reduced cell; all of the angles are ≥90°)†	
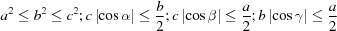	
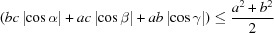	

† Special criteria if equality signs are valid.

**Table 4:** Relations between the cell parameters *a*, *b*, *c*, α , β, γ, the volume *V*, the Laue indices *h*, *k*, *l* and the Miller indices *u*, *v*, *w* of two crystallographic unit cells linearly transformed by the matrix **N**

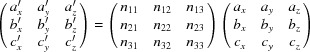	
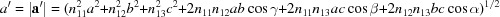	
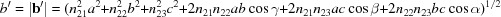	
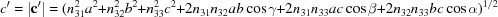	
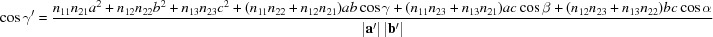	
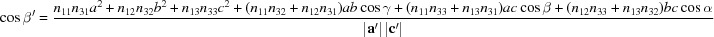	
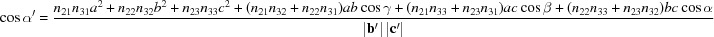	
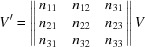	
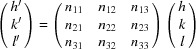	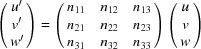

**Table 5:** (*a*) Interchangeability of the Miller indices *u*, *v*, *w* and the crystallographic unit-cell parameters *a*, *b*, *c*, α, β, γ; (*b*) change of sign of the Miller indices *u*, *v*, *w*: effects on the crystallographic unit-cell angles α, β, γ and the Laue indices *h*, *k*, *l*

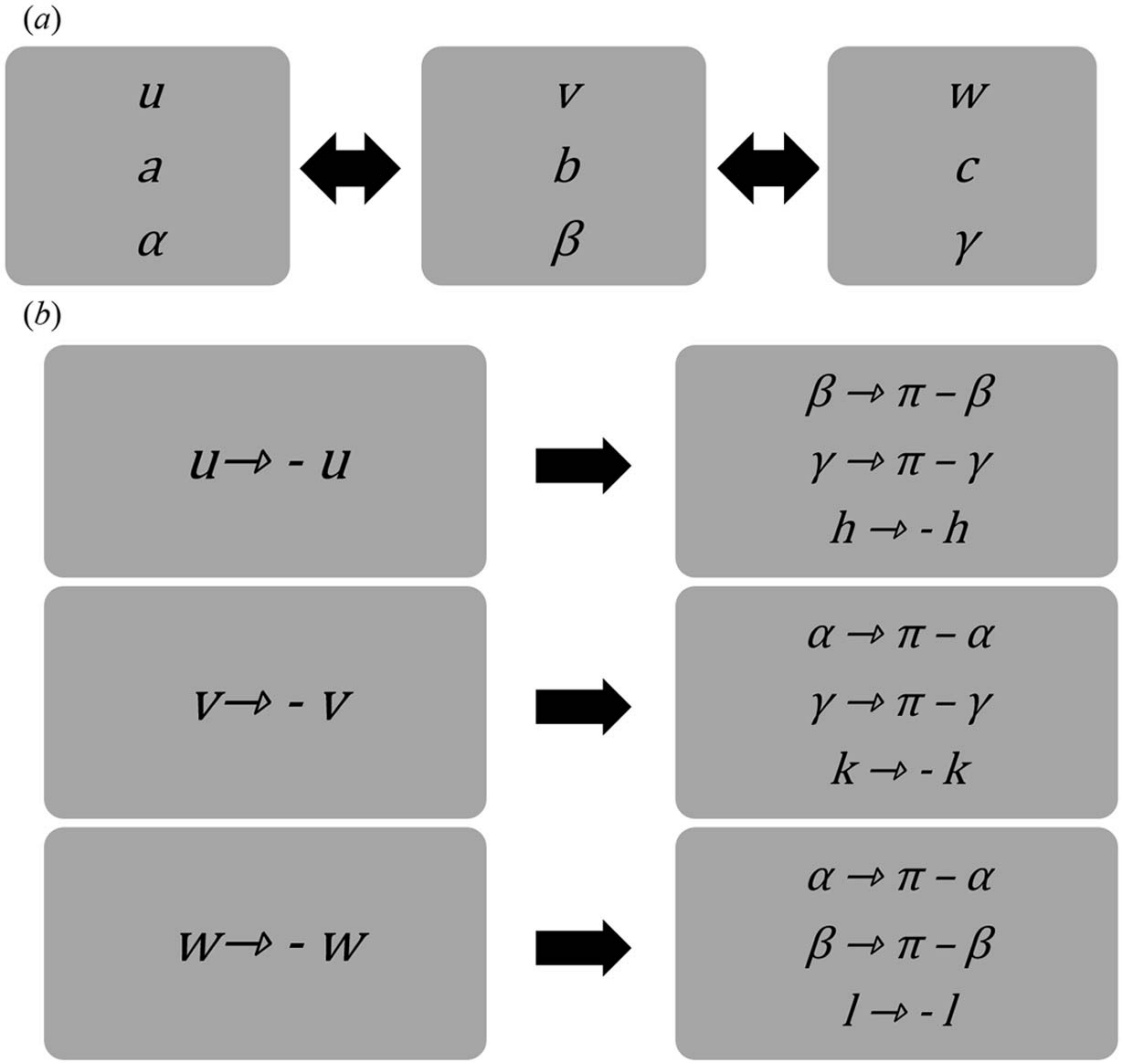

**Table 6:** Indexing of the reciprocal-space map of PQ crystals on HOPG (0001) substrate: corresponding Laue indices of selected individual Bragg peaks for solution 1 (*h*_1_ *k*_1_ *l*_1_) with contact plane (102) and for solution 2 (*h*_2_ *k*_2_ *l*_2_) with contact plane (12−2)

*h*_1_	*k*_1_	*l*_1_	*h*_2_	*k*_2_	*l*_2_
0	0	1	0	1	−1
1	0	1	1	1	−1
1	−1	1	1	0	−2
0	1	2	0	3	−1
0	−1	2	0	1	−3
1	0	0	1	0	0
1	1	0	1	1	1
1	−1	0	1	−1	−1
0	1	1	0	2	0
0	−1	1	0	0	−2
0	1	0	0	1	1

**Table 7:** Unit-cell vectors for the parameters *a*, *b*, *c*, α, β, γ, the Laue indices *h*, *k*, *l* and the Miller indices *u*, *v*, *w* and including the specular scan (*g*_spec_) for the non-rotated (*a*) and the rotated (*b*) case

(*a*) Non-rotated case (*u* = *v* = 0):		
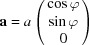		
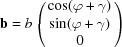		
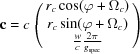	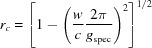	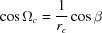
(*b*) Rotated case:		
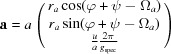	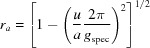	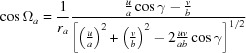
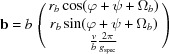	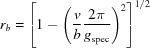	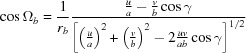
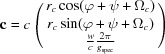	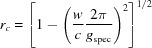	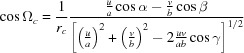
	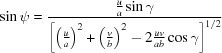	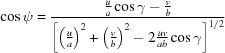

## Appendix A Alternative notation of the rotation matrix

If *u* and *v* have to be assumed to be real numbers (and if not *u* = *v* = 0) it may be more convenient to rewrite equation (14) as

where  and . Then equations (19) and (20) are rewritten as

with  and . From equations (36) and (37) the following expression can be derived:

which comprises the Laue indices *h* and *k*, the three unit-cell parameters *a*, *b*, γ and the rotation angles  and .

## Appendix B Mathematical procedure for analytically determining the cell parameters *a*, *b* and γ

For analytically determining the unit-cell parameters *a*, *b* and γ, it is convenient to introduce the parameters ,  and  with the substitutional relations

Note that  and  are always positive. Using these substitutions, equation (24) can be rewritten as

From three independent Bragg peak series, the parameters ,  and  can be determined by solving the following set of equations:

where

and *i* = 1, 2 and 3. For obtaining *a*, *b* and  from equations (39) to (41) the following identity is helpful:

## Appendix C Compact expressions for the reciprocal cell parameters *a**, *b**and *c**

If *hv* − *ku* = 0 and *u* ≠ 0 equations (22) and (24) can be reduced to

and

respectively. Combining equations (48) and (49) the following expression can be obtained:

If *hv* = *ku* and *v* ≠ 0 the following relation can be deduced in an analogous way:

For *hw* = *lu* and *kw* = *lv* analogous formulas for *b** and *a**, which contain only one real unknown besides the integer variables, are valid.

## Appendix D Useful expressions for determining the Laue indices

From equation (42) the following expression for the Laue index *k* can be derived:

The included components are given in equations (6), (40) and (41). As a term under a root sign must not be negative the following conditions need to be satisfied:

Note that analogous conditions are valid for *k* and *l* with the proper parameters *v*, *b* and *w*, *c*, respectively. Furthermore, rough lower limits for the lattice parameters *a*, *b* and *c* with ,  and  result.

## Appendix E General rotation and analysis for three-component scattering vectors

In the general case, when the individual components of the reciprocal vector *g_x_*, *g_y_* and *g_z_* have to be considered, equation (18) has to be expanded by including an additional rotation around the [0, 0, 1] axis applying the matrix **R**(φ):

which performs a rotation counterclockwise by an angle . Then the reciprocal vector

can be expressed as

From equation (55), using equation (4), it follows that

With

equation (56) can be equivalently expressed as

where **a**, **b** and **c** are the rotated unit-cell vectors with the relations |**a**| = *a*, |**b**| = *b*, |**c**| = *c*, **a**·**b**/(|**a**||**b**|) = cosγ, **a**·**c**/(|**a**||**c**|) = cosβ and **b**·**c**/(|**b**||**c**|) = cosα. These vectors can be written explicitly as

with

Thus, if three reciprocal vectors **g**_1_, **g**_2_ and **g**_3_ are given, the following relation is valid:

where

and ( are the corresponding triples of Laue indices with

Equation (68) can be equivalently expressed as

Furthermore, as

the following relation for the determinants of **G** and **H** is valid:

If the specular scan *g*_spec_ is known it is convenient to apply equations (15)–(17) in the rotation matrix  [equation (35)]. Then the unit-cell vectors **a**, **b** and **c** can be expressed as given in Table 7 ▸. Note that the *z* components are only a function of the Miller index and *g*_spec_.

The unit-cell vectors must be solutions to all reciprocal vectors **g**_*i*_, which, according to equation (58) and comprising equation (71), can be written as

where

and

Therefore, if **a**′, **b**′ and **c**′ are vectors of a superlattice with

where **N** is the transformation matrix [see equations (26)–(29)], the following relation with  can be derived from equation (74):

From equation (71) it can be deduced that 2π**G** ^−1^ **m**, the product of the inverse matrix of three reciprocal vectors with a vector **m**, consisting of a triple of arbitrary integers (*m*_1_, *m*_2_, *m*_3_), leads to a vector of the reduced cell, if **m** matches (*h*_1_, *h*_2_, *h*_3_)^T^, (*k*_1_, *k*_2_, *k*_3_)^T^ or (*l*_1_, *l*_2_, *l*_3_)^T^. If a transformation matrix **N** exists so that **m** equals **N**(*h*_1_, *h*_2_, *h*_3_)^T^, **N**(*k*_1_, *k*_2_, *k*_3_)^T^ or **N**(*l*_1_, *l*_2_, *l*_3_)^T^ a vector of a superlattice is obtained. According to equation (73) it is favourable to select three reciprocal vectors whose matrix results in a determinant, which is as small as possible but unequal to zero. Otherwise a unit cell which is too small and does not satisfy equation (74) for all reciprocal vectors may result. The Buerger and subsequently the reduced cell is obtained by choosing the three shortest vectors which are not coplanar and whose scalar products with all reciprocal vectors yield integers.
